# Alterations of the pancreas in type 1 diabetes – from prior to diagnosis to long-standing disease

**DOI:** 10.1007/s12020-025-04338-0

**Published:** 2025-07-12

**Authors:** Nathalia Guarienti Missima, Henrik Hill, Casian-Simon Aioanei, Per Liss, Daniel Espes

**Affiliations:** 1 Science for Life Laboratory, Department of Medical Sciences, Uppsala University, Uppsala, Sweden; 2 Department of Women´s and Children´s Health, Uppsala University, Uppsala, Sweden; 3 Science for Life Laboratory, Department of Medical Cell Biology, Uppsala University, Uppsala, Sweden; 4 Department of Surgical Sciences, Radiology, Uppsala University, Uppsala, Sweden

**Keywords:** Type 1 diabetes, Exocrine pancreas, Imaging, Volume, Exocrine atrophy

## Abstract

**Purpose:**

In type 1 diabetes (T1D), the loss of insulin-producing beta-cells is the hallmark pathophysiological alteration. However, volumetric and functional abnormalities of the exocrine pancreas are also observed. These changes may result from the loss of insulin’s anabolic effects or reflect an underexplored aspect of T1D. Imaging techniques have enabled a better characterization of pancreatic morphology throughout T1D progression. This study examines exocrine pancreatic alterations at various stages of T1D using CT scans, including assessments conducted prior to diagnosis.

**Methods:**

The study utilized retrospective abdominal CT scans and clinical data collected from Uppsala University Hospital, including 150 T1D subjects, with 15 examined before diagnosis, and 61 age- and gender-matched non-diabetic controls. Volume segmentation and 3D reconstruction assessed the exocrine pancreas, and pancreas volume index (PVI) calculations were standardized using body weight, BMI, and body surface area (BSA). Descriptive and laboratory data were obtained from electronic medical records.

**Results:**

Pancreas volume was significantly reduced in T1D patients. The reduction was more pronounced in patients diagnosed before the age of 20. No significant volume difference was noted in patients before their T1D diagnosis compared to controls, however, a reduction was observed post-diagnosis. Pancreas volume correlated negatively with disease duration and HbA1c levels and correlated positively with body surface area and plasma amylase levels.

**Conclusion:**

Pancreas volume reduction is a consistent feature in T1D, correlating with both disease duration and markers of metabolic control. These findings support the potential of using imaging techniques as a non-invasive method for monitoring T1D progression.

## Introduction

Type 1 diabetes (T1D) is an autoimmune disorder characterized by the destruction of pancreatic islet beta-cells, which leads to hyperglycemia, resulting in metabolic dysfunction that requires lifelong insulin administration. While symptomatic onset typically occurs during childhood or adolescence, symptoms can manifest at a much later age [1]. A reduction in pancreas size has been observed in individuals with long-standing T1D [2, 3]. Nonetheless, at diagnosis, a notable reduction in pancreatic size (20–50%) and exocrine pancreatic insufficiency, ranging from subclinical to symptomatic, are frequently observed and may start even before the onset of islet autoimmunity [4]. Concurrent with reductions in pancreas size, alterations in pancreas shape have been observed [5, 6]. Since pancreatic islets only constitute 1–2% of the pancreatic volume, the mechanisms underlying exocrine loss in T1D remain poorly understood. The hypothesis of potential causes includes developmental defects [7], deficiencies in endocrine-exocrine cross-talk [8] and even an autoimmune and inflammatory destruction of exocrine cells [9]. A recent study in adults with newly diagnosed T1D has described a decrease in pancreatic volume within the first year after diagnosis [10]. A similar study has indicated that, at disease onset, the pancreatic volume was smaller in individuals who ultimately progressed to stage 3 T1D compared to those who remained in stages 1 or 2 [11]. In the context of most systemic diseases, the diagnostic challenge could typically be addressed through the acquisition of a tissue biopsy. However, in the case of T1D, this approach is not feasible due to the considerable risks associated with pancreatic biopsies. Therefore, the development of a dependable non-invasive imaging modality is immensely valuable for monitoring individuals in the pre-symptomatic phases of T1D. Such a tool could provide crucial insights into the risk of progression towards overt disease. Incorporating assessments of exocrine size could enhance the predictive capacity for disease onset and aid in the identification of individuals likely to respond favourably to disease therapies. Nevertheless, research in this area remains nascent. Moreover, exocrine pancreatic insufficiency is often overlooked in individuals with T1D, and there is a paucity of individualized treatment protocols.

The precise monitoring of beta-cell mass is a highly sought-after objective, yet it remains a formidable challenge to achieve, as previously indicated [12]. However, recent advancements offer promising prospects [13]. Instead, monitoring alterations of the whole pancreas could perhaps serve as a surrogate marker with the main advantage that this could be achieved with imaging modalities (i.e. CT or MRI) available in routine clinical care. With this objective in consideration, we conducted a retrospective study with the aim of determining pancreatic alterations in various stages of T1D, including pre-diagnosis, based on retrospective CT scans.

## Research Design and Methods

### Ethical statement

The presented study is based on retrospective data collected at Uppsala University Hospital, Sweden. The study was conducted in accordance with the declaration of Helsinki and approved by the Swedish Ethical Review Authority (Dnr 2019/391).

### Identification of study participants

Patients with T1D catalogued in the Picture Archiving and Communication System (PACS) at Uppsala University Hospital up to 2022 were eligible for study inclusion. Included CT exams were performed between 2010 and 2022 with slice thicknesses ranging from 0.625 to 5.0 mm (most commonly 3.0 mm). All qualifying abdominal CT exams were anonymized and transferred from clinical PACS to research PACS. The included CT exams were initially ordered based on clinical needs and not as part of a research study and therefore the applied CT protocol varies. Exclusion criteria included secondary DM, T2D/LADA, pancreatic tumors or surgery, aortic coarctation surgery, pancreatitis, pancreatic insufficiency, vasculitis, sclerosing cholangitis, pancreas/islet transplantation, anatomical abnormalities, incomplete pancreas images or poor image quality.

Of 344 identified T1D patients, n = 122 lacked abdominal CT scans, n = 25 had no contrast-enhanced CT, n = 11 had suboptimal or incomplete images of the pancreas, and n = 36 were excluded due to fulfilling one or more exclusion criteria. This resulted in a total number of n = 150 T1D patients for analysis. Within this group, n = 15 were analyzed with abdominal CTs prior to the diagnosis of T1D, out of which n = 7 also had undergone a CT scan after the T1D diagnosis. Control subjects (n = 61), matched by age at CT examination, were recruited from patients who had abdominal CT scans at Uppsala University Hospital between 2010 and 2022. They were selected after applying the same exclusion criteria as the T1D cohort, matching in gender distribution and age. For each CT, data on contrast phases (portal, arterial, venous, delayed) and slice thickness were collected from radiological records.

### Descriptive clinical data

Descriptive clinical data including gender, age at onset of T1D, disease duration, body weight and height were collected from electronic medical records. Additionally, laboratory measurements were obtained when available in close temporal proximity to the examination, including HbA1c (reference value 27–42 mmol/mol), C-peptide (reference value 0.4–1.5 nmol/L) and amylase (reference value 0.15–1.1 µkat/L). History of pancreatic tumors, exocrine pancreatic insufficiency, prior pancreatic surgery, and other pertinent information relevant to subject inclusion or exclusion from the study were corroborated using both CT reports and electronic medical records.

### Assessment of pancreas volume

For each CT exam, a radiologist with over ten years of abdominal imaging experience reviewed each contrast phase dataset available, selecting the one that best depicted the pancreas for segmentation. When datasets with different slice thicknesses were available, the one with the thinnest slices was chosen. Manual segmentation was performed by outlining the pancreatic parenchyma on each slice using the LiveWire tool in the Philips Vue PACS, ensuring vessels were not included to prevent volume overestimation. Pancreas volume was automatically calculated, and 3D reconstructions were generated based on the segmentation data. The maximal pancreas length was measured from 3D reconstructed images in the axial plane, with each pancreatic segment measured separately. Widths of the head (including the uncinate process), body, and tail were measured from the axial slices used for segmentation. Anatomical landmarks guided segmentation: the head located within the duodenum curve, right of the superior mesenteric vein (SMV), with the uncinate process as its leftward, caudal extension; the body and tail located behind the lesser sac and stomach. The body-tail border was defined as halfway between the neck and the pancreas end. The pancreatic neck was identified to the left of the head, anterior to the SMV [14]. To standardize pancreas volume across subjects, a pancreas volume index (PVI) was calculated. Three distinct PVIs were computed by dividing pancreas volume by the subject’s: *1)* body weight, *2)* body mass index (BMI), and *3)* body surface area (BSA), calculated based on the Du Bois method [15].

### Statistical methods

Statistical analyses were performed using RStudio 2023.12.1 and GraphPad Prism 9.3 (GraphPad Software, Boston, MA, USA). Data normality was assessed with the D’Agostino & Pearson test. For normally distributed data, an unpaired two-tailed t-test was used; non-normal data were analyzed with the Mann-Whitney test. Pancreas volume comparisons among non-diabetic subjects, T1D patients, and pre-diagnosis individuals were done using the Kruskal-Wallis test with Dunn’s post hoc test. Correlations were assessed with Spearman’s rank. Data are presented as means ± SEM, with statistical significance set at p < 0.05.

## Results

**Fig. 1:**
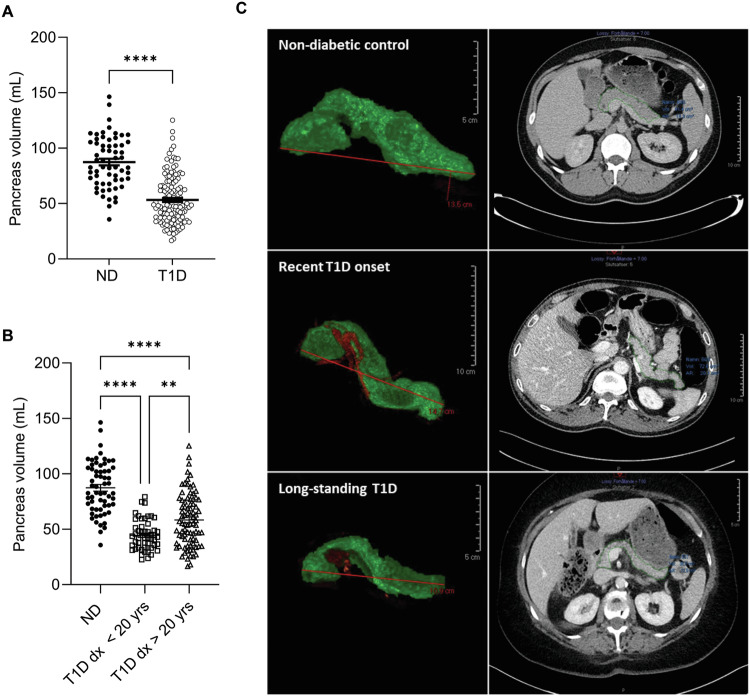
Pancreas volume reduction in non-diabetic and T1D patients. **A** Quantitative analysis of pancreas volume reduction, comparing non-diabetic controls with T1D patients. **B** Subdivision of T1D patients by age at diagnosis (≥20 years), showing differential effects on pancreas volume. **C** Representative 3D reconstructed and CT images of pancreas volumes for non-diabetic controls, recent onset T1D, and long-standing T1D subject. The pancreas is delineated using a continuous green line in the CT images. Data are presented as mean ± SEM. Statistical significance levels are indicated as **p < 0.01 and ****p < 0.0001

The T1D patients and non-diabetic controls were well-matched for age, gender distribution and BMI (Table 1). Pancreas volume was reduced in T1D patients compared to non-diabetic controls (Fig. 1A). Representative images of the pancreas in non-diabetic controls and individuals with recent onset and long-standing T1D are shown in Fig. 1C. This reduction in pancreas volume remained consistent when the PVI was calculated based on body weight, BMI, and BSA (Table 1). When subdividing the T1D group based on age at diagnosis, we found that pancreas volume was smaller in those diagnosed before the age of 20 (n = 51) compared to both non-diabetic subjects and patients diagnosed with T1D at age 20 or later (n = 84) (Fig. 1B). However, it is important to note that there is substantial overlap in pancreas volume between non-diabetic and T1D subjects, indicating that pancreas volume alone cannot be used to distinguish T1D subjects from non-diabetic controls. Our findings show that not only is pancreas volume reduced in T1D subjects, but also the length of the pancreas and the width of each sub-segment (head, body, and tail) (Table 1).

**Table 1:** Pancreas volume in non-diabetic and subjects with type 1 diabetes

Parameter	Non-diabetic (n = 61)	T1D (n = 135)
Age (years)	51.3 ± 2.7 *(range 14 – 74)*	51.8 ± 1.5 *(range 14 – 92)*
Gender (n female, %)	29 (48%)	66 (49%)
Age at T1D onset (years)	*N/A*	28.5 ± 1.6 *(range 1 - 70)*
Disease Duration (years)	*N/A*	23.1 ± 1.4 *(range 0 – 68)*
Height (cm)	172 ± 1.3 *(MD)*	173 ± 0.8 *(MD)*
Body weight (kg)	77.8 ± 2.4 *(MD)*	76.0 ± 1.4 *(MD)*
BMI (kg/m^2^)	25.9 ± 0.7 *(MD)*	25.3 ± 0.4 *(MD)*
Body surface area (m^2^)	1.9 ± 0.03 *(MD)*	1.9 ± 0.02 *(MD)*
Pancreas volume (mL)	87.4 ± 3.0	53.2 ± 1.9****
PVI BW	1.14 ± 0.04	0.71 ± 0.02****
PVI BMI	3.4 ± 0.1	2.1 ± 0.1****
PVI BSA	45.6 ± 1.5	27.9 ± 0.9****
Pancreas length 3D axial	14.2 ± 0.3	13.2 ± 0.2**
Pancreas head width (AP)	3.2 ± 0.1	2.7 ± 0.05****
Pancreas body width (AP)	2.7 ± 0.1	2.1 ± 0.05****
Pancreas tail width (AP)	2.4 ± 0.1	1.7 ± 0.05****

For non-diabetic controls, height was missing for n = 3 patients and body weight for n = 13, and hence, BMI and body surface area could not be calculated for those subjects. Among the participants with type 1 diabetes, height, body weight, BMI, and body surface area were missing for n = 1. Comparisons for pancreas volume and associated metrics were computed with the Mann-Whitney rank test since pancreas volume in patients with type 1 diabetes did not pass the D´Agostino-Pearson omnibus normality test. Participant descriptive parameters were compared with a t-test

All values are presented as mean ± SEM

*MD* missing data

**denotes p < 0.01 and ****p < 0.0001

**Fig. 2:**
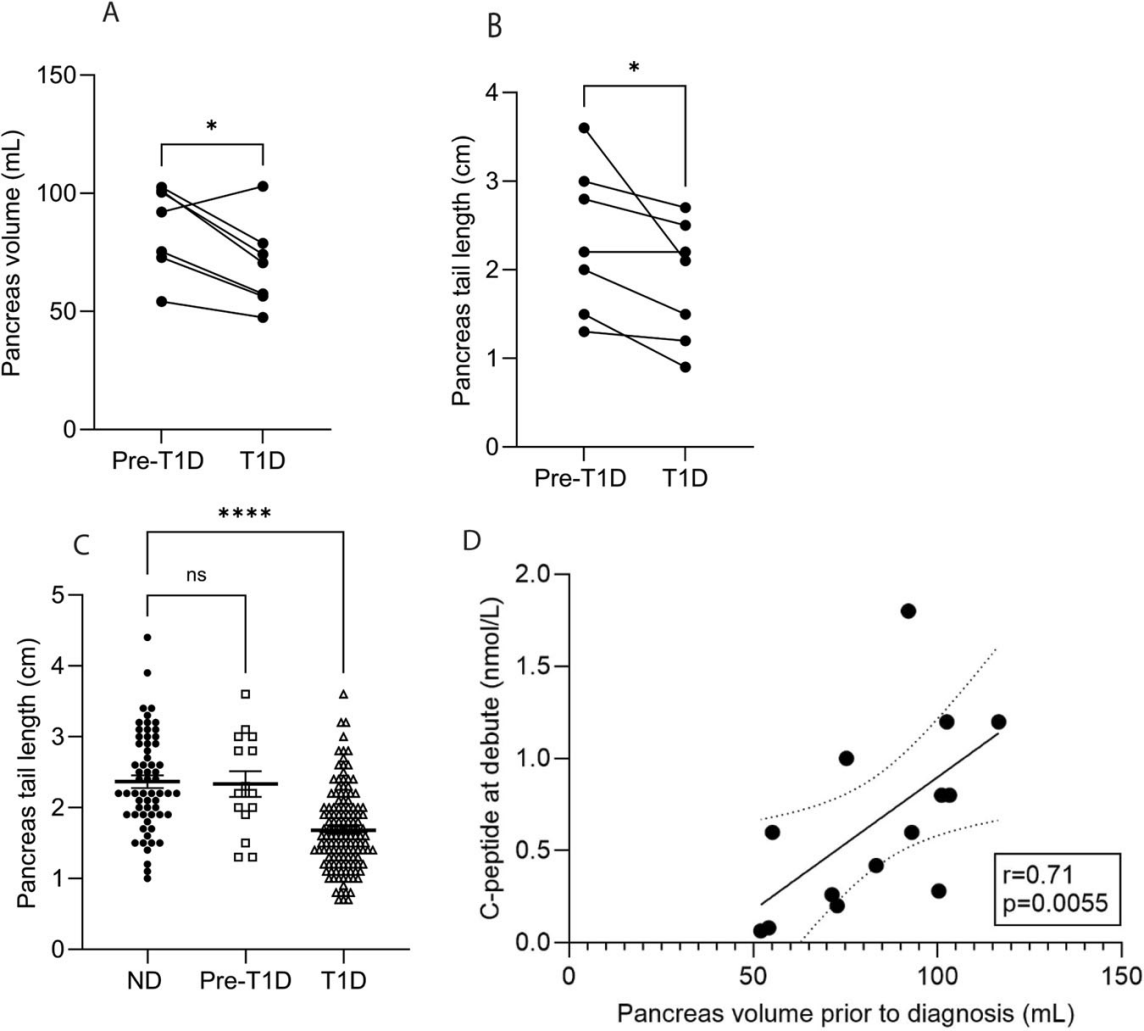
Alterations of pancreas volume and length prior to and after diagnosis of type 1 diabetes. **A** Pancreas volume and **B** tail length reduction in T1D patients compared to pre-diagnosis. **C** Pancreas tail length reduction in non-diabetic, pre-T1D, and T1D individuals. **D** Pancreas volume before diagnosis correlates with C-peptide levels at diagnosis. Reference value for C-peptide 0.4 – 1.5 nmol/L. Data presented as mean ± SEM, with corresponding statistical significance levels indicated (*p < 0.05, ****p < 0.0001)

We identified n = 15 T1D patients who had undergone a CT scan prior to their diagnosis (ranging from −1 to −19 years). The pancreas volume in this cohort was not significantly different from that of non-diabetic controls. This finding also held true for those scanned within five years of their T1D diagnosis (n = 8) (data not shown). However, among these 15 patients, there were seven who had undergone repeated scans both before and after T1D diagnosis, and in these patients, we observed a reduction in pancreas volume. Specifically, six of the seven patients displayed a reduction in volume and tail length, while the pancreas volume increased in one patient (Fig. 2A). Of the other examined pancreatic parameters (i.e. length and width of sub-regions), we found that the width of the pancreas tail was reduced post-diagnosis (Fig. 2B), whereas other measures remained unchanged. Notably, all patients showed a reduction in width except one, whose width remained unchanged. Interestingly, this was not the same patient who exhibited increased volume (Fig. 2B). Additionally, we found that pancreas volume prior to diagnosis strongly correlated with C-peptide levels at diagnosis (r = 0.71, p = 0.0055) (Fig. 2D).

**Fig. 3:**
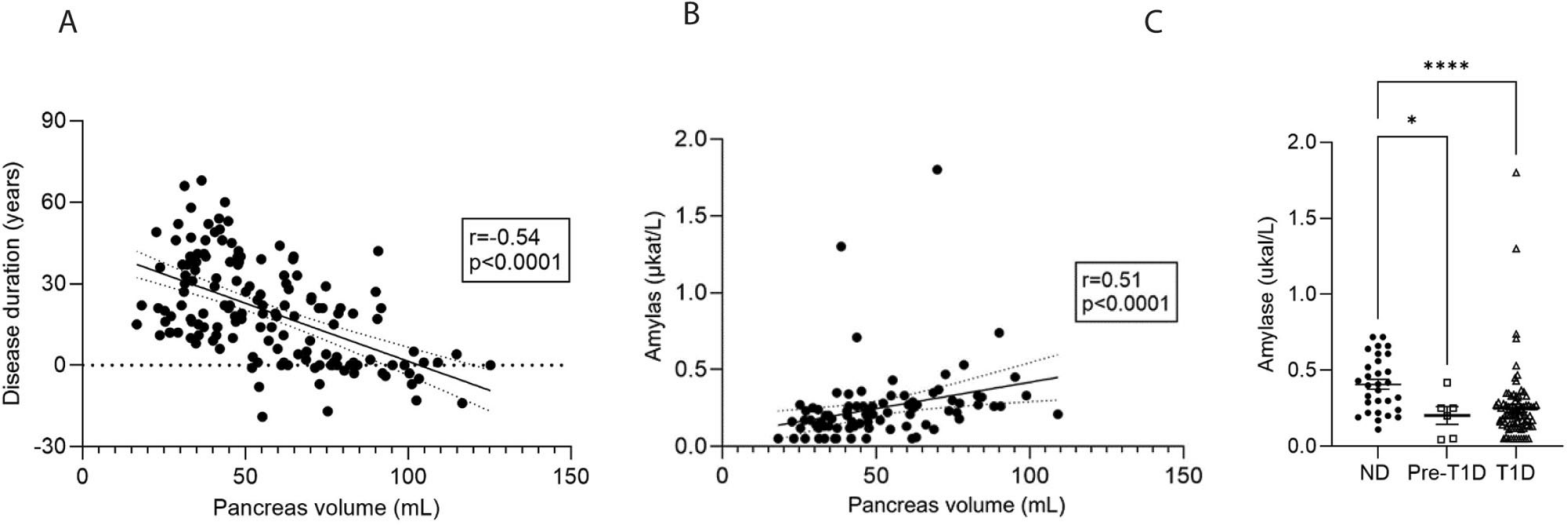
Correlation of pancreas volume with disease duration and functional measures. **A** negative correlation was observed between disease duration and pancreas volume in established T1D. **B** Plasma amylase levels correlate positively with pancreas volume in T1D. **C** The plasma amylase levels was found to be lower in both pre-diagnosis and established T1D patients compared to controls. Reference value for amylase 0.15 – 1.1µkat/L). Data presented as mean ± SEM, with corresponding statistical significance levels indicated (*p < 0.05, ****p < 0.0001)

When analysing correlations in patients with established T1D, we observed a negative correlation between disease duration and pancreas volume (r = −0.42, p < 0.0001) (Fig. 3A). However, no correlation was found between pancreas volume and age per se in either the T1D group (r = −0.14, p = 0.12) or the non-diabetic group (r = 0.02, p = 0.87). Furthermore, a negative correlation was noted between HbA1c levels and pancreas volume in T1D patients (r = −0.25, p = 0.0047, n = 130, accounting for missing data). The complete set of correlations computed for both T1D patients and non-diabetic individuals is presented in Table 2 and Table 3.

**Table 2:** Correlation of pancreas volume in subjects with established T1D

Parameter	r-value	p-value	n
Age (years)	−0.14	0.12	135
Disease duration (years)	−0.42	**<0.0001**	134
Age at onset (years)	0.34	**<0.0001**	134
Height (cm)	0.28	**0.0013**	134
Body weight (kg)	0.31	**0.0003**	134
BMI (kg/m^2^)	0.20	**0.023**	134
Body surface area (m^2^)	0.33	**<0.0001**	134
HbA1c (mmol/mol)	−0.25	**0.0047**	130
Amylase (µkat/L)	0.51	**<0.0001**	89
Pancreas length 3D (axial)	0.53	**<0.0001**	135
Pancreas head width (AP)	0.48	**<0.0001**	135
Pancreas body width (AP)	0.55	**<0.0001**	135
Pancreas tail width (AP)	0.54	**<0.0001**	135

Correlations were computed based on non-parametric Spearman correlation, number of available data points are depicted per each parameter since retrospective data were missing for some of the parameters (especially amylase levels).

Bold values indicates statistical significant *P* values.

**Table 3:** Correlation of pancreas volume in non-diabetic controls

Parameter	r-value	p-value	n
Age (years)	0.02	0.87	61
Height (cm)	0.42	**0.001**	58
Body weight (kg)	0.60	**<0.0001**	48
BMI (kg/m^2^)	0.38	**0.0068**	48
Body surface area (m^2^)	0.59	**<0.0001**	48
HbA1c (mmol/mol)	−0.26	0.66	6
Amylase (µkat/L)	0.026	0.89	30
Pancreas length 3D (axial)	0.61	**<0.0001**	61
Pancreas head width (AP)	0.48	**<0.0001**	61
Pancreas body width (AP)	0.58	**<0.0001**	61
Pancreas tail width (AP)	0.47	**<0.0001**	61

Correlations were computed based on non-parametric Spearman correlation, number of available data points are depicted per each parameter since retrospective data were missing for some of the parameters (especially HbA1c and amylase levels).

Bold values indicates statistical significant *P* values.

Among the measures, BSA displayed the strongest correlation with pancreas volume in both T1D patients (r = 0.33, p < 0.0001) and non-diabetic individuals (r = 0.59, p < 0.0001). Notably, despite the absence of clinical diagnosis of exocrine insufficiency in the electronic medical records, a correlation was observed between plasma amylase levels and pancreas volume (r = 0.51, p < 0.0001, n = 89, accounting for missing data) in the T1D cohort (Fig. 3B). Conversely, among non-diabetic controls, where amylase measurements were available for n = 30 subjects, no correlation was found between pancreas volume and amylase levels (r = 0.026, p = 0.89). Based on the available data, we found that amylase levels were reduced in both the pre-diagnosis group and the T1D group compared to the control group (Fig. 3C).

## Discussion

Our findings show that the pancreas is smaller in both long-standing and new-onset T1D patients compared to age-matched non-diabetic subjects. We found that pancreas volume is negatively correlated with disease duration in our T1D cohort, spanning from 0 to 68 years. The reduction of pancreas volume in T1D has been reported in several previous studies [16–19], although other studies have found no correlation between disease duration and pancreas volume [10, 20, 21]. The progression of T1D varies depending on the age at onset, with older age at diagnosis being associated with a slower rate of C-peptide decline (i.e. loss of endogenous beta-cell mass) [22]. However, pancreas volume was not correlated with age per se in T1D patients, suggesting that the volume reduction is a component of the disease process rather than a reflection of ageing. This notion is further supported by the lack of correlation between pancreas volume and age in the non-diabetic subjects. In a previous study, we observed that pancreas volume persistently decreased during the first two years after T1D diagnosis, providing additional evidence that alterations in the exocrine pancreas are part of the T1D disease process [23]. Additionally, a recent study utilizing longitudinal MRI scans in a larger cohort of patients with new-onset T1D demonstrated a decrease in pancreas volume following diagnosis, with the most significant reduction occurring within the first year [6]. Furthermore, another recent study demonstrated that alterations in the exocrine pancreas are evident even before the clinical onset of T1D (i.e. in stages 1 and 2) [11]. Previous studies using CT have shown that pancreas volume increases during childhood and adolescence until the age of 20, after which it remains stable during adulthood and tends to decrease from age 60 onwards in non-diabetic individuals [24]. While T1D is most frequently diagnosed in the pediatric population, it is estimated that approximately 40% of patients develop T1D as adults [25, 26]. It can be speculated that if T1D negatively affects the exocrine pancreas, the impact would be more pronounced if the onset of T1D occurs during childhood when the pancreas is still growing. In our dataset, we found that T1D patients diagnosed before the age of 20 had a smaller pancreas compared to both non-diabetic subjects and those diagnosed with T1D at age 20 or later. This could potentially increase the risk of exocrine dysfunction. Since we don´t systematically screen for exocrine dysfunction in Sweden, our retrospective clinical material lacks data on this aspect. Nonetheless, we observed a correlation between plasma amylase levels and pancreas volume, even though none of the included patients had a clinical diagnosis of exocrine pancreatic insufficiency. This condition is likely underdiagnosed, given the prevalence reported in most studies with targeted testing [27]. In individuals with T1D, pancreatic tissues exhibit pronounced acinar atrophy and angiopathy alongside reduced presence of intralobular adipocytes. Furthermore, autopsy studies of the pancreas from patients with T1D demonstrate heightened periductal and perivascular fibrosis. These alterations underscore distinctive morphological variations within the exocrine pancreas, discernible across ageing processes or upon the onset of T1D [28]. Notably, exocrine pancreas alterations in T1D are not solely anatomical but most likely also functional. Recent evidence suggested that lipase and trypsinogen levels were significantly reduced in individuals with two or more autoantibodies (i.e. stage 1–2), recent-onset T1D and established T1D compared to control subjects and those with a single autoantibody (i.e. stage 0), whereas amylase levels were reduced only in those with established T1D [29]. Furthermore, our observations aligned with previous findings, showing a significant reduction in systemic amylase levels in both the pre-T1D and T1D groups compared to controls.

Despite evidence suggesting a reduction in pancreas volume before the diagnosis of T1D, we did not observe a smaller pancreas volume in subjects examined before their T1D diagnosis. However, it should be noted that the number of identified subjects was limited, and the time to diagnosis ranged from −1 to −19 years. Also, our study identified the patients retrospectively and were not part of a defined cohort of autoantibody-positive individuals. Therefore, these results should be interpreted cautiously, especially considering the overlap in pancreas volume observed among patients with long-standing T1D and non-diabetic subjects. Among those who had been examined both prior- and post-diagnosis, we did observe a reduction in pancreas volume in all but one subject. Furthermore, when specifically analyzing different anatomical regions of the pancreas, we found that the tail width was reduced in patients examined before and after diagnosis. This finding could potentially represent an early marker to monitor longitudinally in the early stages of T1D.

Interestingly, we also found a negative correlation between pancreas volume and HbA1c levels in patients with T1D. Previous studies on new-onset patients have shown a negative correlation between the low blood glucose index (LBGI) and pancreas volume, but no correlation with other markers of metabolic control [11]. In our cohort, the negative correlation with HbA1c could partly be explained by disease duration, but these two parameters were not on their own correlated (r = −0.07, p = 0.46). Correlating alterations in the exocrine pancreas with the reduction of beta-cell mass would be highly valuable. Unfortunately, C-peptide (a surrogate marker of the functional beta-cell mass) is not routinely included in the follow-up of T1D patients in Sweden and was, therefore, only available at diagnosis for most patients.

Based on the results of this study and the current literature, if imaging is to be used as a diagnostic tool for identifying individuals at risk of progressing to clinical onset of T1D, it should not be compared to the volume or metrics of other individuals. Instead, it should serve as a longitudinal measure within patients. Ideally, imaging modalities could serve as an objective measure in combination with the presence of autoantibodies and functional measures of beta-cell function. Recently, the CD3-directed monoclonal antibody Teplizumab demonstrated success in delaying the progression towards manifest T1D and was approved by the FDA as the first-in-class therapy for this purpose [30]. This marks the inception of a prospective era wherein a plethora of treatment options may soon be accessible for both impeding the progression towards manifest T1D and mitigating the destruction of beta-cells in individuals with recently diagnosed T1D. Similar publications have presented compelling evidence of the beneficial effects of Teplizumab even post-diagnosis of T1D [31], and we have also recently disseminated data endorsing allogeneic mesenchymal stromal cells (MSCs) therapy in new-onset T1D for beta-cell preservation [32]. In the new era of beta-cell preserving therapies, which ideally would be initiated prior to clinical symptoms of T1D, imaging techniques could become a valuable tool for identifying the individuals who would benefit mostly from interventions and the timing for initiating such therapies. Interestingly, there is data to support the notion that even first-degree relatives of patients with T1D have a smaller pancreas volume when compared to healthy subjects without a family history of T1D [33].

A key limitation of the current study is the lack of standardisation and the absence of relevant laboratory measurements, such as amylase and C-peptide, which were not included in the original clinical evaluations. Additionally, variability in imaging protocols exists, as most scans were conducted for non-pancreatic indications. Many T1D patients and control subjects with native CT scans were excluded due to segmentation challenges. Without contrast enhancement, distinguishing pancreatic parenchyma from surrounding tissues is difficult, potentially leading to overestimation of pancreatic volume, especially when patients were not fasted prior to imaging. In summary, our findings indicate that pancreas volume and associated measures are diminished in patients with both new-onset and long-standing T1D, with pancreas volume exhibiting a negative correlation with disease duration. Furthermore, even in the absence of clinically established exocrine insufficiency, pancreas volume correlates with plasma levels of amylase, suggesting not only anatomical but also functional alterations in the exocrine pancreas in T1D. Although we cannot demonstrate a reduction in pancreas volume long before the onset of T1D, our results reveal a clear reduction in pancreatic size during the transition towards manifest T1D.

## Data Availability

The data that support the findings of this study are not openly available due to reasons of sensitivity and are available from the corresponding author upon reasonable request.
